# Ion Transport Modulators as Antimycobacterial Agents

**DOI:** 10.1155/2020/3767915

**Published:** 2020-11-20

**Authors:** Steven C. Mitini-Nkhoma, Narmada Fernando, G. K. D. Ishaka, Shiroma M. Handunnetti, Sisira L. Pathirana

**Affiliations:** ^1^Institute of Biochemistry, Molecular Biology and Biotechnology, University of Colombo, No: 90, Cumaratunga Munidasa Mawatha, Colombo 3, Sri Lanka; ^2^Malawi-Liverpool-Wellcome Trust Clinical Research Programme, P.O. Box 30096, Chichiri, Blantyre 3, Malawi; ^3^Australian College of Business and Technology, 442 Galle Road, Colombo 3, Sri Lanka

## Abstract

There is an urgent need for better and safer therapeutic interventions for tuberculosis (TB). We assessed the effects of FDA-approved ion transport modulators, namely, ambroxol HCl, amiloride HCl, diazoxide, digoxin, furosemide, hydrochlorothiazide (HCTZ), metformin, omeprazole, pantoprazole, phenytoin, verapamil, and drug X and Y on the growth of free and intracellular *Mycobacterium bovis* BCG. Free and intracellular *M. bovis* BCG were cultured in the presence or absence of the test drugs for 3 to 9 days and then quantified. For both free and intracellular bacteria, cultures that were exposed to furosemide, phenytoin, or drug Y yielded lower bacteria counts compared to drug-free controls (*p* < 0.05). The same was observed with diazoxide, HCTZ, verapamil, and drug X, but only for intracellular *M. bovis* BCG (*p* < 0.05). To assess the effects of the drugs on bactericidal activity of rifampicin, free and intracellular *M. bovis* BCG were treated with rifampicin alone or in combination with each of the thirteen test drugs for 3 to 9 days. For extracellular bacteria, higher bacteria clearance rates were observed in cultures exposed to rifampicin in combination with amiloride HCl, diazoxide, digoxin, furosemide, HCTZ, metformin, pantoprazole, phenytoin, drug X, or drug Y than those exposed to rifampicin alone, indicating that rifampicin had a synergistic effect with these test drugs. Rifampicin was also synergistic with ambroxol HCl, diazoxide, digoxin, furosemide, HCTZ, omeprazole, pantoprazole, phenytoin, verapamil, and drug X against intracellular *M. bovis* BCG. The antimycobacterial properties exhibited by the ion transport modulators in this study make them viable candidates as adjuncts to the current anti-TB regimens.

## 1. Introduction

Tuberculosis (TB) remains the leading infectious cause of death even though it is now curable. At present, TB can only be cured using drug regimens comprising at least 3 compounds administered over no less than 6 months [1]. Patients at times fail to adhere to the 6-month long course of antibiotics. As poor drug adherence is one of the main drivers of antibiotic resistance, developing shorter therapeutic regimens for TB would help reduce the burden of multidrug-resistant (MDR) TB.

As developing drugs *de novo* is a long and expensive process, drug repurposing and repositioning have garnered attention as cost-effective strategies for reducing the burden of TB [2]. At present, several FDA-approved ion transport modulators including verapamil and lansoprazole are among the most promising potential anti-TB agents [3, 4]. However, only a few ion transport modulators have been assessed for potential anti-TB activity. In this study, we assessed the antimycobacterial properties of thirteen FDA-approved ion transport modulators, namely, ambroxol HCl, amiloride HCl, diazoxide, digoxin, furosemide, hydrochlorothiazide (HCTZ), metformin, omeprazole, pantoprazole, phenytoin, verapamil, and drugs X and Y. The authors have withheld the identities of drug X and drug Y pending further studies.

## 2. Materials and Methods

### 2.1. Drugs

All drugs were purchased from Sigma-Aldrich (St Louis, MO, USA). Stock solutions were prepared in dimethylsulfoxide (DMSO), aliquoted, and stored at -20°C for no more than six weeks. Working solutions were prepared in Middlebrook 7H9 broth or RPMI. All drugs were used at concentrations that were below their respective maximum free plasma concentrations when administered to humans at therapeutic doses (see Table 1). We also assessed the toxicity of the test drugs to THP-1 derived macrophages using the sulforhodamine B assay as described previously [5], and all the test drugs were only used in subsequent experiments at concentrations that were not toxic to the macrophages (Figure S1 in Supplementary Materials).

### 2.2. THP-1 and *Mycobacterium bovis* BCG Culture

THP-1 is a human leukaemia monocytic cell line that is extensively used to study macrophage physiology [6]. THP-1 cells were cultured in RPMI 1640 media supplemented with 10% foetal bovine serum (FBS), 2 mM glutamine, 10 mM HEPES, 4500 mg/l glucose, 1500 mg/l sodium bicarbonate, and 0.05 mM 2-mercaptoethanol. Prior to each experiment, the cells were differentiated into macrophages by treatment with 200 nM phorbol myristate acetate (PMA) for 3 days. All reagents which were used for culturing THP-1 cells were purchased from Sigma-Aldrich.

*Mycobacterium bovis* BCG-1 (Russia) was grown in Middlebrook 7H9 broth with 10% oleic acid, albumin, dextrose, and catalase (OADC) growth supplement and 0.05% Tween 80.

### 2.3. Assessment of the Effects of the Test Drugs on the Growth of Extracellular *M. bovis* BCG

*M. bovis* BCG was seeded into 96 well plates at 0.2 × 10^6^ colony forming units (CFUs) in 200 *μ*l per well. The cultures were treated with the test drugs for 3, 6, or 9 days at 37°C, after which viable bacteria were quantified using the BacTiter-Glo™ microbial cell viability assay kit following manufacturer's instructions.

### 2.4. Assessment of the Effects of the Test Drugs on the Growth of Intracellular *M. bovis* BCG

THP-1 derived macrophages were cultured as described above. The macrophages were then infected with *M. bovis* BCG at a multiplicity of infection (MOI) of 10 and incubated at 37°C for one hour. Non-internalised bacteria were removed by washing with PBS and quantified as described above. The amount of internalised bacteria was calculated by subtracting the amount of non-internalised bacteria from the amount of bacteria that was originally added to the macrophages. Next, the cultures were treated with the test drugs and incubated for 3, 6, or 9 days at 37°C. Bacteria were then recovered from the macrophages by lysis with 0.2% saponin for 10 minutes [7]. The lysate was washed twice with PBS, after which the bacteria were quantified.

### 2.5. Assessment of the Effects of the Test Drugs on Antimycobacterial Activity of Rifampicin

Free or intracellular *M. bovis* BCG was exposed to rifampicin (2000 ng/ml) alone or in combination with each of the test drugs for 3, 6, or 9 days, after which the bacteria were quantified.

### 2.6. Data Analysis

Statistical analyses and graphical presentation were performed using GraphPad Prism version 8.4.2 (GraphPad Software, La Jolla, California, USA) and R version 3.5.1 (R Core Team, Vienna, Austria). Bacteria load between different cultures was compared using Kruskal-Wallis nonparametric analysis of variance (ANOVA) followed by Dunn's multiple comparisons post hoc analysis.

## 3. Results and Discussion

### 3.1. Ion Transport Modulators Inhibit Growth of *M. bovis* BCG

**Figure 1:**
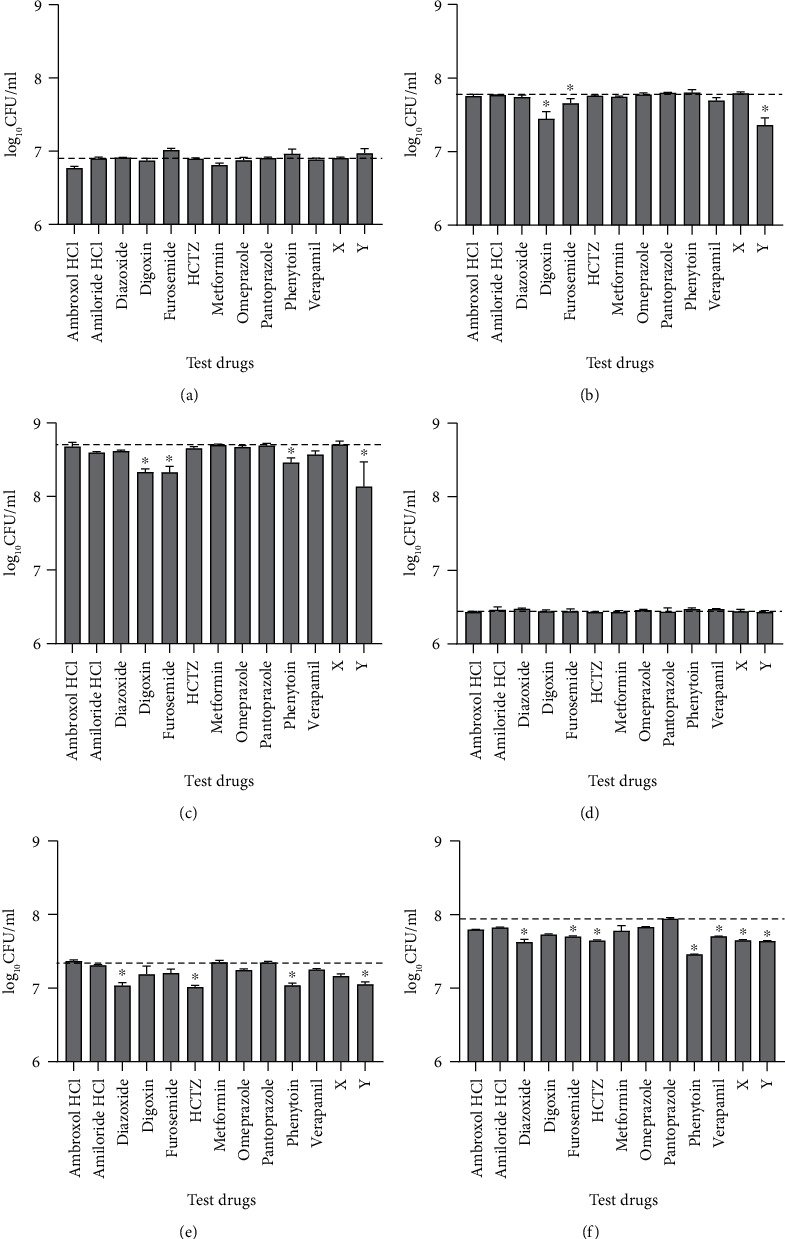
Median (+IQR) bacterial concentration (colony forming units/ml) after exposure of extracellular (a–c) and intracellular (d–f) *M. bovis* BCG to different test drugs for 3 (a, d), 6 (b, e), or 9 (c, f) days. Dashed lines indicate the CFU in drug-free controls. ^∗^ indicates *p* < 0.05 vs. drug-free controls. HCTZ: hydrochlorothiazide.

Following treatment of 0.2 × 10^6^ extracellular *M. bovis* BCG with different test drugs for 3, 6, or 9 days, at least 1.1 × 10^6^ viable bacteria were recovered from each of the cultures, indicating that none of the drugs were bactericidal at the tested concentrations. No significant differences in viable bacteria counts were observed between any of the drug-treated cultures and drug-free controls after three days of treatment (Figure 1(a)). Bacterial cultures that were treated with digoxin, furosemide, or drug Y for 6 or 9 days had significantly lower bacterial counts compared to drug-free controls (*p* < 0.05), indicating that these drugs are bacteriostatic to extracellular *M. bovis* BCG (Figures 1(b) and 1(c)). Significantly lower bacterial counts were observed in cultures that were treated with phenytoin only after treatment for 9 days (*p* < 0.05).

Following infection of 0.2 × 10^6^ macrophages with *M. bovis* BCG at MOI 10, an average of 0.505 × 10^6^ bacteria (25.2%) were taken up by the macrophages, and the rest were washed out of the wells with PBS. When the intracellular bacteria were treated with the test drugs for 3, 6, or 9 days, ≥0.507 × 10^6^ bacteria were recovered from each culture, indicating that none of the test drugs were bactericidal to the intracellular bacteria at the concentrations that were used. Exposure of the cultures to the test drugs for 3 days did not yield any significant difference in bacteria counts between the drug-treated samples and drug-free controls (Figure 1(d)). However, cultures that were treated with diazoxide, HCTZ, phenytoin, or drug Y for 6 or 9 days had significantly lower bacteria counts than drug-free controls (*p* < 0.05) (Figures 1(e) and 1(f)). Cultures that were exposed to furosemide, verapamil, or drug X for 9 days also had significantly lower bacterial counts than drug-free controls (*p* < 0.05) (Figure 1(f)).

In this study, diazoxide, HCTZ, verapamil, and drug X were bacteriostatic to intracellular, but not to extracellular bacteria. One possible explanation for this is that the macrophages might have accumulated the drugs, particularly in the compartments harbouring the *M. bovis*. Alternatively, the drugs might have enhanced control of *M. bovis* by the macrophages. While diazoxide, HCTZ and drug X have not been previously reported to enhance host antimycobacterial responses, various authors have previously demonstrated that verapamil enhances autophagy and control of Mtb in primary human and mouse macrophages [8–11]. While TB and other infectious diseases are traditionally treated with agents that directly compromise pathogen physiology, there is growing interest in host-directed therapy; the control of pathogens by potentiating the host's antipathogen responses [12]. In addition to small molecules, other potential host-directed therapeutic strategies for TB include cytokine therapy and therapeutic vaccines [12].

In addition to enhancing control of mycobacteria by macrophages, verapamil is bactericidal to extracellular Mtb at concentrations higher than those that were used in this study [10]. While ambroxol and metformin did not have a significant effect on the growth of *M. bovis* BCG in this study, they have both been shown to enhance the control of mycobacteria by macrophages at concentrations higher than those that were employed in this study [13, 14].

To the best of our knowledge, this is the first time that diazoxide, digoxin, furosemide, HCTZ, phenytoin, and drugs X and Y have been shown to have antimycobacterial activity.

### 3.2. Ion Transport Modulators Enhance Antimycobacterial Activity of Rifampicin

**Figure 2:**
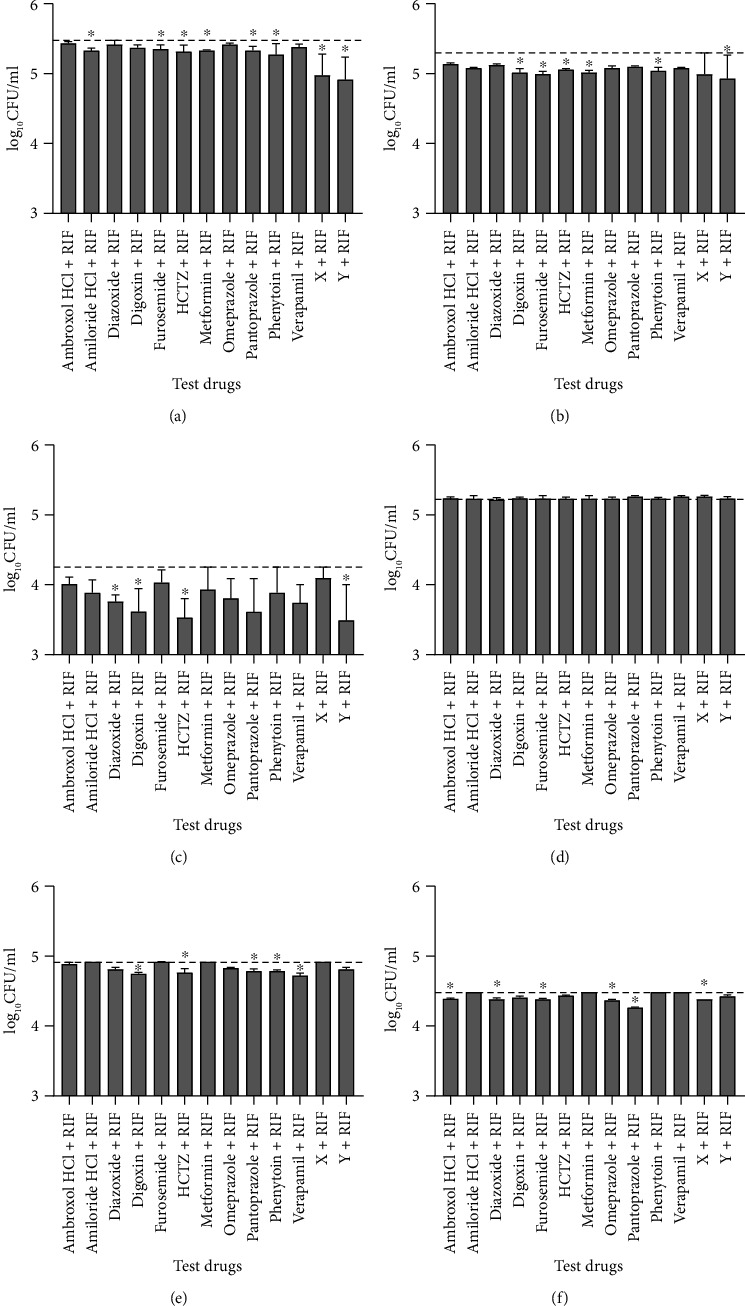
Median (+IQR) bacterial concentration (colony forming units/ml) after exposure of extracellular (a–c) and intracellular (d–f) *M. bovis* BCG to rifampicin (RIF) alone or in combination with different test drugs for 3 (a, d), 6 (b, e), or 9 (c, f) days. Dashed lines indicate CFU in cultures treated with rifampicin alone. ^∗^ indicates *p* < 0.05 vs. cultures treated with rifampicin only. HCTZ: hydrochlorothiazide.

Following treatment of extracellular bacteria with rifampicin alone or in combination with each of the test drugs for 3, 6, or 9 days, the viable bacteria count was higher in cultures that were treated with rifampicin alone than in cultures that were treated with rifampicin in combination with HCTZ or drug Y at all time points. Cultures that were treated with amiloride HCl, diazoxide, digoxin, furosemide, metformin, pantoprazole, phenytoin, or drug X in combination with rifampicin had lower bacteria counts than cultures treated with rifampicin alone on at least one time point (Figures 2(a)–2(c)).

For intracellular bacteria, there were no differences in bacteria counts between cultures that were treated with rifampicin alone or in combination with any of the test drugs for 3 days (Figure 2(d)). However, cultures that were treated with ambroxol HCl, diazoxide, digoxin, furosemide, HCTZ, omeprazole, pantoprazole, phenytoin, verapamil, or drug X in combination with rifampicin had significantly lower bacterial counts than those treated with rifampicin alone on at least one time point (*p* < 0.05) (Figures 2(e) and 2(f)).

Our findings are consistent with the findings of the previous studies which showed that verapamil and metformin enhance the antimycobacterial activity of rifampicin and other first-line anti-TB drugs [8, 15, 16]. Verapamil enhances the activity of rifampicin at least in part by interfering with both host and bacterial efflux pumps [8]. Host and bacterial efflux pumps reduce the concentration of anti-TB drugs inside macrophages and bacteria, respectively, leading to drug tolerance [17]. This allows the bacteria to persist for longer, necessitating the use of prolonged treatment regimens to eliminate Mtb. In addition, overactive bacterial efflux systems are responsible for a proportion of the cases of MDR-TB [17]. Combining anti-TB drugs with some ion transport modulators with efflux pump activity could therefore help reduce the burden of MDR-TB.

## 4. Conclusions

In summary, we have shown that furosemide, phenytoin, and drug Y are bacteriostatic to both extracellular and intracellular *M. bovis* BCG at physiologically achievable concentrations. In addition, diazoxide, HCTZ, verapamil, and drug X are bacteriostatic to intracellular *M. bovis* BCG, while digoxin is bacteriostatic to extracellular *M. bovis* BCG.

The results also show that diazoxide, digoxin, furosemide, HCTZ, pantoprazole, phenytoin, and drug X enhance the bactericidal activity of rifampicin against both extracellular and intracellular *M. bovis* BCG. Amiloride HCl, metformin and drug Y enhance the activity of rifampicin against extracellular *M. bovis* BCG, while ambroxol HCl, omeprazole and verapamil enhance the activity of rifampicin against intracellular *M. bovis* BCG.

As this study was done *in vitro* using *M. bovis* BCG and an immortalized cell line, our findings may not accurately predict how the drugs would perform *in vivo*. Therefore, there is a need to investigate the efficacy of the ion transport modulators used in this study against mycobacteria *in vivo*.

## Figures and Tables

**Table 1:** Concentrations of the drugs tested in the current study and their maximum free plasma concentrations in humans.

Drug	Indications	Concentration used in the current study (ng/ml)	Maximum free plasma concentration in ng/ml [reference]
Ambroxol HCl	Bronchiectasis, emphysema	6	6 [18]
Amiloride HCl	Hypertension, CHF^∗^	40	40 [19]
Diazoxide	Hypoglycaemia	5000	5000 [20]
Digoxin	AF^⸸^, CHF	2	2 [21]
Furosemide	Hypertension, CHF	500	500 [22]
HCTZ	Hypertension	500	642 [23]
Metformin	Diabetes mellitus	1000	1000 [24]
Omeprazole	Gastritis, peptic ulcer disease	200	200 [25]
Pantoprazole	Gastritis, peptic ulcer disease	180	180 [26]
Phenytoin	Epilepsy	2000	2000 [27]
Verapamil	Hypertension	24	24 [28]
Drug X		2	2
Drug Y		2	2

^∗^Congestive heart failure. ^⸸^Atrial fibrillations.

## Data Availability

The data used to support the findings of this study is included in the supplementary materials.

## Supplementary Materials

Supplementary Materials Figure S1: mean (+SD) viability of THP-1 derived macrophages following exposure to different concentrations of test drugs. Table S1: effects of test drugs on intracellular and extracellular *M. bovis* BCG following treatment for 3, 6, or 9 days. Table S2: effects of test drugs on antimycobacterial activity of rifampicin against intracellular and extracellular *M. bovis* BCG following treatment for 3, 6, or 9 days.
