# Supplementary material for: Ion Transport Modulators as Antimycobacterial Agents
Source: Tuberc Res Treat. 2020 Nov 20;2020:3767915. doi: 10.1155/2020/3767915 (PMC7700046; doi:10.1155/2020/3767915)
Supplement: Supplementary Materials — Figure S1: mean (+SD) viability of THP-1 derived macrophages following exposure to different concentrations of test drugs. Table S1: effects of test drugs on intracellular and extracellular M. bovis BCG following treatment for 3, 6, or 9 days. Table S2: effects of test drugs on antimycobacterial activity of rifampicin against intracellular and extracellular M. bovis BCG following treatment for 3, 6, or 9 days. [file 3767915.f1.zip › Figure S1.pdf]

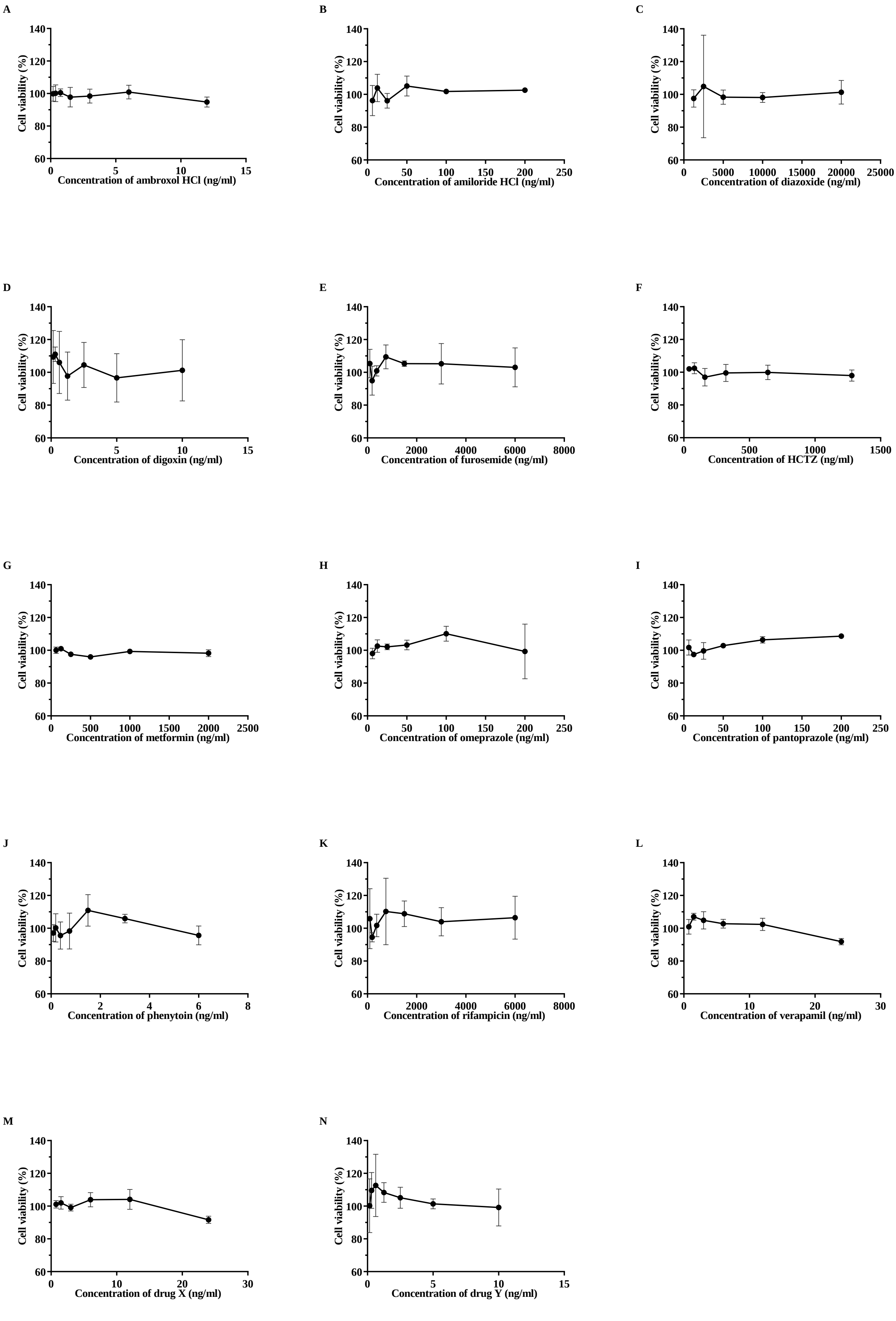


**Figure S1: Mean (+SD) viability of THP-1 derived macrophages following exposure to different concentrations of test drugs. HCTZ, hydrochlorothiazide**
