# Supplementary material for: Ion Transport Modulators as Antimycobacterial Agents
Source: Tuberc Res Treat. 2020 Nov 20;2020:3767915. doi: 10.1155/2020/3767915 (PMC7700046; doi:10.1155/2020/3767915)
Supplement: Supplementary Materials — Figure S1: mean (+SD) viability of THP-1 derived macrophages following exposure to different concentrations of test drugs. Table S1: effects of test drugs on intracellular and extracellular M. bovis BCG following treatment for 3, 6, or 9 days. Table S2: effects of test drugs on antimycobacterial activity of rifampicin against intracellular and extracellular M. bovis BCG following treatment for 3, 6, or 9 days. [file 3767915.f1.zip › Table S1.pdf]

Table S1: Effects of test drugs on intracellular and extracellular *M. bovis* BCG following treatment for 3, 6 or 9 days

Drug	CFU/ml for extracellular bacteria on day 3	CFU/ml for extracellular bacteria on day 6	CFU/ml for extracellular bacteria on day 9	CFU/ml for intracellular bacteria on day 3	CFU/ml for intracellular bacteria on day 6	CFU/ml for intracellular bacteria on day 9
Ambroxol HCl	5528250	58042690	454939645	2726710	24239755	61586965
Ambroxol HCl	6868505	59968060	458912250	2718405	22716320	62670565
Ambroxol HCl	5819700	51891770	490317565	2706525	22891090	62311115
Ambroxol HCl	5983620	52392315	464198600	2616855	20879345	65537675
Ambroxol HCl	5703565	56352445	544932860	2627715	24235565	62738815
Ambroxol HCl	5984450	60794540	546622000	2894830	23775680	62497665
Amiloride HCl	7663925	59020795	369459195	2539425	20229985	66327275
Amiloride HCl	7603165	59148795	405401595	3345525	20603205	67262475
Amiloride HCl	7773455	59590395	418559995	3129335	20595730	66865575
Amiloride HCl	8410650	56883195	338687995	2674175	19823375	64999375
Amiloride HCl	8318710	59052795	389836795	2890030	16515900	64385740
Amiloride HCl	8160410	57619195	399001595	2929005	21652700	69230090
Diazoxide	8218775	57439995	433561595	2661890	12554955	41799625
Diazoxide	8284330	58412795	424396795	2720525	11677390	41328790
Diazoxide	8244355	53375995	423526395	2998500	11066050	40855325
Diazoxide	8168405	59283195	404940795	3039020	9649250	46092115
Diazoxide	7884585	52339195	400025595	3132825	10555350	46212425
Diazoxide	8168405	50969595	406067195	2995980	9068685	42753115
Digoxin	7739080	4544000	229376000	2608900	19320965	53970265
Digoxin	7651135	35263995	259071995	2821030	21698600	52839675
Digoxin	7275375	26944000	212480000	2903860	1865090	54602540
Digoxin	8754430	27520000	203264000	2928410	16693355	55028575
Digoxin	7003545	34943995	28160000	2724310	14224315	52727240
Digoxin	7179435	28480000	215552000	2689595	13844140	51219090
Drug-Free	9171245	58765185	529334730	2987365	23180030	87800310
Drug-Free	8613050	55064890	482135905	2555165	20816845	83902570
Drug-Free	7534140	83513015	539863135	2857470	22065235	88288205
Drug-Free	7241365	70020610	474733360	2710970	23679465	86208290
Drug-Free	6657845	57899470	523347400	2849215	21568895	110586965
Drug-Free	8751840	58736785	522585390	2839820	21951640	87678105
Furosemide	10649225	45119995	29184000	2800765	12736930	51720725
Furosemide	10545295	5760000	261119995	2758915	14457140	51221365
Furosemide	9002275	38079995	254976000	2583595	16522645	50506400
Furosemide	11752525	46015995	182784000	2732700	17845520	50631000
Furosemide	9218135	52095995	211968000	3077770	18846735	49109200
Furosemide	10193515	54591995	214016000	2970055	15408155	50279950
HCTZ	7996515	58489595	455014395	2720890	10023440	45528965
HCTZ	8050080	58739195	460134395	2633100	9527550	45419240
HCTZ	7619155	57241595	444159995	2631825	10223315	44672075
HCTZ	8476210	54284795	514611195	2699410	11198050	44465490
HCTZ	7814230	57478395	442572795	2713755	10464070	43376815
HCTZ	7866195	56121595	428390395	2788450	10792900	43015700
Metformin	7041585	50955835	516905780	2811485	19565950	54412225

Metformin	6162815	57791255	480339195	2635685	21975415	72767715
Metformin	6240605	55684285	513371110	2688880	23775985	61612075
Metformin	6854890	55842620	504456240	2626695	21195340	53965365
Metformin	6360905	56869310	497136685	2850305	24316150	70643125
Metformin	6575665	54932285	480182780	2708060	22826665	58349290
Omeprazole	8666490	54425595	490239995	2796915	17688115	66638165
Omeprazole	8185995	57177595	493414395	2905765	18244015	66333225
Omeprazole	7677515	54764795	461311995	3023820	18459840	68930140
Omeprazole	7463255	63187195	474419195	2843595	17358050	68194350
Omeprazole	7424080	62604795	462591995	2943120	16817270	69102775
Omeprazole	7489635	62649595	443596795	2750390	17320980	67211200
Pantoprazole	8092455	63116795	500223995	2789965	23117385	86966905
Pantoprazole	7880590	63494395	502271995	3135670	21612140	90437970
Pantoprazole	7982125	62412795	483481595	2667730	22749785	88714930
Pantoprazole	8468215	64838395	603699195	3095735	20983490	83939075
Pantoprazole	7805435	62636795	450201595	2536255	23203690	95510015
Pantoprazole	8277135	61548795	429823995	2608120	20720185	84179980
Phenytoin	9521945	18496000	325119995	3069460	9929540	27365365
Phenytoin	8850370	65087995	364031995	2789875	11037960	29714650
Phenytoin	8106845	73535995	276479995	3102545	11784885	28857065
Phenytoin	14462800	23808000	301567995	2979190	11612295	28391475
Phenytoin	9545930	61951995	235520000	2644080	10800610	28972215
Phenytoin	9026260	68671995	245760000	2961735	10235165	28912100
Verapamil	8034890	55097595	401612795	3024100	17032130	50571065
Verapamil	7560790	54239995	358860795	3019560	18929345	50946350
Verapamil	8070065	52390395	374783995	2733820	18188865	48044850
Verapamil	7473645	46188795	457420795	2752450	17564960	51975875
Verapamil	7472050	41465595	348211195	2918965	18110020	49546875
Verapamil	7911770	43468795	367615995	3074485	16931960	50979340
Drug X	7498430.779	64185594.99	552140794.6	2955786.4	15478041.6	44923462.5
Drug X	7900575.003	62067195.15	512460795	2773256.668	15894592	45512075
Drug X	7902173.986	61926395.16	504063995.1	2985645.32	15380668.8	46534425
Drug X	8807198.364	61644795.18	476518395.3	2755891.04	13705484.8	43632050
Drug X	8189191.435	62489595.12	422041595.9	2774683.072	13386553.6	44873062.5
Drug X	8208379.231	67865594.7	611225594	2484190.8	13160291.2	43754287.5
Drug Y	8210777.705	21567998.32	288255997.2	2796624.628	10930438.4	44174462.5
Drug Y	10889074.23	24447998.09	32767999.68	2962017.8	10518883.2	44323562.5
Drug Y	10657221.7	20287998.42	238591997.7	2603968.92	10613657.6	44386212.5
Drug Y	10881079.32	25023998.05	27647999.73	2754586.24	11626809.6	43562750
Drug Y	7267377.735	40319996.85	317951996.9	2669500.4	11655324.8	43377687.5
Drug Y	7667123.485	19071998.51	30719999.7	2597986.44	13759468.8	43614725
