# Supplementary material for: Ion Transport Modulators as Antimycobacterial Agents
Source: Tuberc Res Treat. 2020 Nov 20;2020:3767915. doi: 10.1155/2020/3767915 (PMC7700046; doi:10.1155/2020/3767915)
Supplement: Supplementary Materials — Figure S1: mean (+SD) viability of THP-1 derived macrophages following exposure to different concentrations of test drugs. Table S1: effects of test drugs on intracellular and extracellular M. bovis BCG following treatment for 3, 6, or 9 days. Table S2: effects of test drugs on antimycobacterial activity of rifampicin against intracellular and extracellular M. bovis BCG following treatment for 3, 6, or 9 days. [file 3767915.f1.zip › Table S2.pdf]

Table S2: Effects of test drugs on antimycobacterial activity of rifampicin against intracellular and extracellular *M. bovis* BCG following treatment for 3, 6 or 9 days

Drug	CFU/ml for extracellular bacteria on day 3	CFU/ml for extracellular bacteria on day 6	CFU/ml for extracellular bacteria on day 9	CFU/ml for intracellular bacteria on day 3	CFU/ml for intracellular bacteria on day 6	CFU/ml for intracellular bacteria on day 9
Ambroxol HCl + Rifampicin	287995	132705	11595	163370	75310	21325
Ambroxol HCl + Rifampicin	279650	132785	6340	180780	70805	23355
Ambroxol HCl + Rifampicin	269025	140970	8830	174475	85100	26195
Ambroxol HCl + Rifampicin	275395	131335	15150	177600	78995	24975
Ambroxol HCl + Rifampicin	276770	128055	11475	184365	69395	24310
Ambroxol HCl + Rifampicin	271185	125360	7455	176170	72790	25110
Amiloride HCl + Rifampicin	213230	117265	9140	180635	76500	27995
Amiloride HCl + Rifampicin	192940	117015	4605	182880	82910	28640
Amiloride HCl + Rifampicin	274080	116750	9325	168915	78695	29545
Amiloride HCl + Rifampicin	215435	109595	4665	158105	80310	30105
Amiloride HCl + Rifampicin	213650	106215	17420	164945	76180	29785
Amiloride HCl + Rifampicin	214195	117365	6480	192490	78200	29595
Diazoxide + Rifampicin	272885	131810	10475	164905	60900	23825
Diazoxide + Rifampicin	251685	119120	5970	170085	62660	23545
Diazoxide + Rifampicin	223765	120500	405	187395	65445	25140
Diazoxide + Rifampicin	300525	130410	5600	165425	56040	24895
Diazoxide + Rifampicin	244350	137630	5995	181960	67040	24700
Diazoxide + Rifampicin	293260	116885	2165	161215	53875	24455
Digoxin + Rifampicin	257185	99310	4255	171175	53695	24850
Digoxin + Rifampicin	227395	98515	3460	177205	53560	25180
Digoxin + Rifampicin	221255	98785	11205	172500	52980	25310
Digoxin + Rifampicin	266555	108090	520	178415	51975	25165
Digoxin + Rifampicin	228505	118280	7815	184955	54400	25650
Digoxin + Rifampicin	251865	96785	4085	183540	51120	25355
Furosemide + Rifampicin	224395	93235	8250	190395	69360	23685
Furosemide + Rifampicin	206050	86480	18745	158085	69500	22730
Furosemide + Rifampicin	198510	92115	2490	183050	78775	25035
Furosemide + Rifampicin	220845	101605	2530	183730	82000	24285
Furosemide + Rifampicin	258515	101955	15435	158855	84035	24555
Furosemide + Rifampicin	273790	83120	13560	160105	79755	23835
HCTZ + Rifampicin	239540	104305	3060	160030	49060	26585
HCTZ + Rifampicin	208550	108010	6100	181200	62550	26210
HCTZ + Rifampicin	209355	105470	3115	158660	56185	26640
HCTZ + Rifampicin	190205	113155	2575	170305	57880	27235
HCTZ + Rifampicin	189955	112975	3610	175350	62890	26585
HCTZ + Rifampicin	301210	100945	6620	160380	54365	26045
Metformin + Rifampicin	218330	101310	3850	166675	76175	28835
Metformin + Rifampicin	210260	105785	1400	176515	80725	28595
Metformin + Rifampicin	238195	105085	19215	188995	70630	28690
Metformin + Rifampicin	200950	94435	17025	164320	80200	28865
Metformin + Rifampicin	199475	96630	13125	192145	81515	29535
Metformin + Rifampicin	212280	91650	3340	176150	79130	29625
Omeprazole + Rifampicin	268075	121445	6220	171935	63485	23785
Omeprazole + Rifampicin	275560	121570	9515	180645	65240	22155
Omeprazole + Rifampicin	252785	117580	6670	174720	67655	23820

Omeprazole + Rifampicin	257330	103620	19025	158970	67075	23865
Omeprazole + Rifampicin	255880	111370	5040	180305	64835	22110
Omeprazole + Rifampicin	239745	99000	1790	192600	65340	23055
Pantoprazole + Rifampicin	250750	123950	2335	162835	56335	18385
Pantoprazole + Rifampicin	237670	129410	5870	157970	62345	18375
Pantoprazole + Rifampicin	224325	122990	1980	192460	59135	17870
Pantoprazole + Rifampicin	196990	109410	9860	184970	57930	18265
Pantoprazole + Rifampicin	177810	113735	840	176100	60675	17820
Pantoprazole + Rifampicin	168160	109535	19805	192260	63725	17990
Phenytoin + Rifampicin	161320	104795	11970	168945	60090	30735
Phenytoin + Rifampicin	176740	110560	16705	160205	60035	30360
Phenytoin + Rifampicin	193960	85615	2050	183860	57950	30125
Phenytoin + Rifampicin	268495	84305	3415	163240	59765	30475
Phenytoin + Rifampicin	262140	104595	1500	176815	64285	31115
Phenytoin + Rifampicin	162505	132235	19835	180200	61250	29750
Rifampicin	299080	169875	19310	161810	75195	29605
Rifampicin	308360	172150	22205	171625	85355	30160
Rifampicin	307380	169495	25855	172000	82500	30405
Rifampicin	315155	175630	9655	161430	70320	30355
Rifampicin	299500	143030	11990	192360	86385	28935
Rifampicin	292205	184425	15745	168205	72900	29000
Verapamil + Rifampicin	266085	110240	3860	185105	46995	30060
Verapamil + Rifampicin	253720	106810	6870	191300	54595	30815
Verapamil + Rifampicin	240110	110695	5885	191485	47100	30875
Verapamil + Rifampicin	235405	118190	5350	180075	56825	29920
Verapamil + Rifampicin	229500	119545	19755	181880	52980	30210
Verapamil + Rifampicin	227745	119435	1945	165375	46200	30295
X + Rifampicin	227846	114910	16865	186536	84777	23242
X + Rifampicin	231792	106550	4427	188602	70618	23034
X + Rifampicin	263308	109265	16622	174661	80688	23509
X + Rifampicin	231091	121770	6370	170075	77193	24059
X + Rifampicin	219938	121009	7759	167677	81297	24089
X + Rifampicin	191682	105346	20474	185877	73942	24110
Y + Rifampicin	256467	104747	11330	165399	60461	27003
Y + Rifampicin	158982	100094	508	170575	62706	27637
Y + Rifampicin	152959	98761	1230	164611	66110	27114
Y + Rifampicin	182560	108628	9756	174585	59947	28016
Y + Rifampicin	196140	96759	1821	176879	61263	25198
Y + Rifampicin	167869	120718	4326	169773	68569	27134
